# Analyzing Left-Truncated Samples with the Cox Model in the Presence of Missing Covariates

**DOI:** 10.1007/s12561-024-09442-9

**Published:** 2024-07-02

**Authors:** Omar Vazquez, Hayley M. Locke, Sharon X. Xie

**Affiliations:** Department of Biostatistics, Epidemiology and Informatics, University of Pennsylvania, Philadelphia, PA USA

**Keywords:** Left truncation, Missing covariates, Cox regression, Augmented inverse probability weighting, Multiple imputation

## Abstract

Delayed enrollment of subjects into a time-to-event study may result in a sample with biased outcome and covariate distributions. Additionally, missing covariate data may arise in these studies when information is difficult to collect due to patient burden or high testing costs. Some common missing data strategies, such as multiple imputation (MI) and augmented inverse probability weighting (AIPW), involve modeling the distribution of the missing covariate, which may be inaccurate with a left-truncated sample. Through simulation studies, we explore the performance of these methods in estimating Cox regression parameters under a variety of truncation and missing data scenarios. We find that MI and AIPW may be approximately unbiased when truncation is very low. Otherwise, biased estimation of the covariate distribution can cause MI to perform poorly. Similarly, AIPW may rely more heavily on correct estimation of the probability of having non-missing covariates in some settings. We apply these approaches to a Parkinson’s disease dementia biomarker study. In analyzing left-truncated data with missing covariates, careful consideration of the data characteristics and method assumptions is needed to obtain valid results.

**Supplementary Information:**

The online version contains supplementary material available at 10.1007/s12561-024-09442-9.

## Introduction

Left truncation often arises in studies of chronic disease progression with time-to-event outcomes, which are defined as the time from some initiating event (time 0) to the event of interest. Under left truncation, subjects come under observation after time 0 by study design. For example, we consider a cohort study that aims to identify biomarkers associated with cognitive decline in Parkinson’s disease (PD). This study enrolled cognitively normal PD patients and followed them until they experienced cognitive symptoms. Time of PD diagnosis was selected as time 0 and was retrospectively ascertained. This design excludes any subjects who experience cognitive symptoms prior to when they would have entered the study. As a result, the sample is left truncated. To avoid truncation, one could select study enrollment as time 0. However, this outcome would be less clinically relevant and more difficult to interpret than time from PD diagnosis to cognitive symptom onset.

**Fig. 1:**
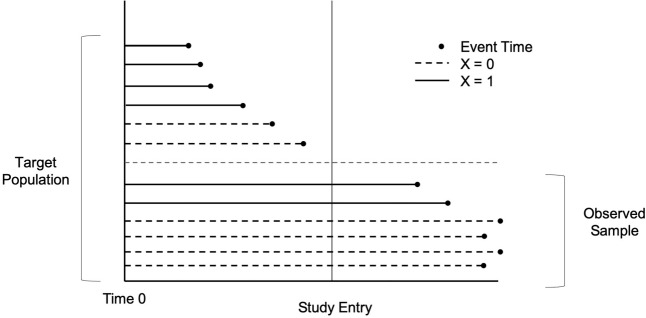
Hypothetical Example of Covariate Bias Due To Left Truncation. Participants with a covariate value of X=1 tend to have shorter event times and are more likely to experience the event prior to study entry, making them ineligible for the study. As a result, there is a discrepancy between the sample proportion of X (1/3) and the underlying population proportion (1/2)

In the above example, patients who experience cognitive symptoms shortly after being diagnosed with PD are less likely to be eligible to participate in the study. As a result, larger event times are over-represented in the sample. The sample covariate distribution is similarly affected by left truncation, as it has a higher frequency of values associated with longer event times [4, 5]. Consequently, unadjusted estimation of the failure time and covariate distributions may be biased. A hypothetical example of this phenomenon with a binary covariate X is illustrated in Fig. 1. X = 1 is associated with shorter failure times. As a result, these subjects are less likely to be sampled. In the population, half of individuals have X = 1. However, the biased sampling scheme distorts this proportion so that only one-third of sampled subjects have X = 1.

The analysis of left-truncated samples can be further complicated by missing covariate data. Baseline covariates may be missing purely due to chance or by study design, particularly if they are invasive or expensive to collect. Simply ignoring subjects with missing values can be inaccurate and inefficient when the complete case subset is not representative of the whole sample. A few general missing data methods are widely used and applicable to many types of outcome models. One strategy, referred to as inverse probability weighting (IPW) [19], is to weight each observation in the complete case subset to make this subset more representative of the larger sample. Alternatively, one may predict missing values directly, which is the main idea behind multiple imputation (MI) [17]. Augmented inverse probability weighting (AIPW) [16, 18] combines elements of MI and IPW to achieve greater robustness. While these approaches have been studied in the Cox model with right-censored data [23, 25], less attention has been devoted to left-truncated and right-censored data. Hu et al. [7] proposed IPW estimators for length-biased data, where entry times are assumed to be uniformly distributed. Shen and Cook [21] developed an EM-algorithm-based approach to address missing binary covariates in a left-truncated sample. This method accounts for the difference between the sample and population covariate distributions under the biased sampling scheme. It also requires specification of the baseline hazard with a fixed number of parameters. Currently, we are not aware of any studies that have explored IPW, MI, and AIPW when applied to the semiparametric Cox model [6] under left truncation with an unspecified entry time distribution.

In Cox regression with fully observed covariates, one accounts for left truncation by using a modified risk set definition [9]. In this paper, we aim to develop MI and AIPW estimation methods using risk set adjustment to account for left truncation in the presence of missing covariates. Through simulation studies, we evaluate and compare the performance of these two approaches together with complete case analysis and IPW. In particular, we highlight the impact of selection bias on estimating the missing covariate distribution in MI and AIPW. We focus on the case where the missing covariate is binary and complete variables are categorical. This scenario is of interest because the correct imputation model follows a standard distribution and can be exactly specified. With other data types, the imputation model must be approximated. By examining binary missing covariates and categorical fully observed covariates, we can eliminate poor imputation model approximation as a potential source of bias in MI. Any remaining bias can be attributed to the effect of left truncation on estimating the missing covariate distribution, allowing us to better understand method performance. The principles presented in this study will help guide researchers in selecting the appropriate missing data method for their studies.

The rest of this paper is organized as follows. In Sect. 2, we discuss several missing data approaches and their expected performance under left truncation. Simulation studies comparing these methods under a variety of truncation and missing data scenarios are presented in Sect. 3. In Sect. 4, we apply these missing data methods to a study of biomarkers for cognitive decline among Parkinson’s disease (PD) patients. Discussion and concluding remarks are given in Sect. 5.

## Missing Data Methods Under Left Truncation

Consider a time-to-event study with *n* subjects. Let *U* and *C* be the times from time 0 to the event and censoring, respectively. The survival outcome is composed of the observed time $T = \text{ min }(U,C)$ and the event indicator $\delta = I(U \le C)$, where $I(\cdot )$ is the indicator function. Further, let *L* be the time from time 0 to study entry. The analysis model is the Cox model shown in Equation (1), where X is a binary covariate and **W** is a set of binary or categorical fully observed covariates.

1 $$\begin{aligned} h(t \vert \text {X},{{\textbf {W}}}) = h_0(t)\exp (\beta _1 \text {X} + \varvec{\beta }^T_2 {{\textbf {W}}}). \end{aligned}$$

In Equation (1), $h_0(t)$ is the unspecified baseline hazard. Other relevant quantities include the cumulative baseline hazard $H_0(t) = \int _0^t h_0(u)du$ and the covariate-specific survival curve, $S(t \vert \text {X},{{\textbf {W}}}) = \exp (-H_0(t)e^{\beta _1\text {X}+\varvec{\beta }^T_2{{\textbf {W}}}})$. A central idea underlying Cox regression analysis is the risk set. In the usual right censoring case, subjects are at risk if they have not yet experienced the event. Specifically, the risk set at time *t* is the set of all subjects i for which $Y_i(t) = I(t \le T_i) = 1$. To accommodate left truncation, the risk set is adjusted to include only those subjects who have already entered the study by time *t*, so that the at risk indicator at time t becomes $Y^*_i(t) = I(L_i < t \le T_i)$ [2]. This allows for unbiased estimation of the log-hazard ratios $\beta _1$ and $\varvec{\beta }_2$ under the conditional independence assumption $U \perp (L,C) \vert \text {X}, {{\textbf {W}}}, T>L$ [10, 15]. We use the symbol $\perp$ to denote independence. For all missing data methods examined, the outcome model we consider is the Cox regression model using $Y^*_i(t)$ to account for left truncation.

We consider the case where X may be missing for some individuals, but **W** is observed for all in the sample. Let *R* be a binary variable that is equal to 1 when X is non-missing and 0 when X is missing. A key assumption in the analysis of incomplete data is the mechanism underlying missingness. The methods discussed in this study assume that the data are either missing completely at random (MCAR) or missing at random (MAR). Under MCAR, missingness is independent of any observed or unobserved variables in the study. When the data are MAR, missingness is related to the observed variables but is independent of unobserved data [11]. In a regression setting with missing covariates, a further distinction is whether missingness is related to the outcome. Since the analysis conditions on covariates, missingness related to fully observed covariates does not bias estimation of regression coefficients when using the complete case subsample [11]. Similarly, the Cox regression model under left truncation conditions on the value of *L*. As a result, MCAR mechanisms and MAR mechanisms related to fully observed covariates or study entry time have similar properties. For the purposes of this paper, we define MCAR as missingness that is independent of any variable or depends on **W** or *L*. Alternatively, MAR is defined as missingness that depends on the observed survival time *T* or the censoring indicator $\delta$. Importantly, this does not include mechanisms that rely on either the failure time *U* or censoring time *C*. We consider *U*- or *C*-dependent missingness as missing not at random because *U* and *C* are not fully observed (*C* is only known for censored subjects, and *U* is only known for uncensored subjects). Missing not at random is a much more difficult situation to address and is beyond the scope of this article. MCAR missingness might occur if a covariate is measured on a random subset of subjects at baseline, perhaps by study design, while MAR may arise in some specialized study designs, such as case-cohort sampling [13].

### Complete Case Analysis

The simplest missing data method is complete case analysis (CC), in which observations with unobserved values are excluded from analysis. If the data are MCAR, the non-missing subjects are a random subsample of the data, and inference is consistent. However, under an outcome-dependent MAR mechanism, the complete case subset is not representative of the larger sample, and hazard ratio estimation will be biased. Furthermore, removing incomplete observations from the data leads to inefficient inference.

### Inverse Probability Weighting

Under the MAR mechanism, non-missing subjects are more likely to have specific outcome values, creating a biased complete case sample. This bias can be corrected by weighting complete observations in the Cox model according to the probability of X being non-missing, denoted by $\pi _i$ for subject i [19]. The subscript *i* is removed when doing so does not cause ambiguity. This probability is commonly estimated using a logistic regression model with outcome $R_i$. This model must be correctly specified to address bias induced by an MAR mechanism. Similar to CC, IPW ignores missing subjects in the final analysis, generally leading to inefficient estimates.

### Multiple Imputation

Both CC and IPW do not directly incorporate missing subjects into the Cox regression analysis. As a result, some potentially useful information is ignored in the analysis, leading to larger standard errors. MI overcomes this inefficient estimation issue by predicting missing values directly, producing an imputed dataset with no missing values. Then, the Cox model with modified risk sets is applied as if there were no missing data. This procedure is repeated multiple times to account for the variability associated with predicting missing values, resulting in multiple imputed datasets. Rubin’s rules [17] are used to combine these estimates and perform inference.

The imputation process relies on having a closed-form representation for the distribution of X given the fully observed variables $T, \delta$, and ${{\textbf {W}}}$. This is the distribution from which imputed values will be drawn for missing subjects. In discussing conditional distributions of X, we use $p(\cdot )$ to denote the probability mass function of a categorical variable, $f(\cdot )$ to denote the probability density function of a continuous variable, and $P(\cdot )$ to denote probability. White and Royston [25] provide a detailed study of MI to address missing covariates in the Cox model in the absence of left truncation. They demonstrate that if X is binary, **W** is categorical, and $p(\text {X} \vert {{\textbf {W}}})$ is assumed to be a logistic regression model, $p(\text {X} \vert T,\delta ,{{\textbf {W}}})$ is also a logistic regression model with predictors **W**, $\delta$, $H_0(T)$ and the interaction between $H_0(T)$ and **W**. If either covariate is continuous, approximations are required to obtain a closed-form imputation model. For example, if $f(\text {X} \vert {{\textbf {W}}})$ is assumed to be normally distributed with mean $\eta _0 +\varvec{\eta }_1^T{{\textbf {W}}}$ and variance $\sigma ^2$, then $f(\text {X} \vert T,\delta ,{{\textbf {W}}})$ can be approximated by linear regression. However, this approximation fails if $\beta _1^2 \sigma ^2 H_0(T)$ is large. We focus on the binary covariate situation so that any bias that arises is not due to poor approximation of the true imputation model, but potentially due to poor estimation of the imputation model parameters under left truncation. We assume the population distribution of $\text {X} \vert {{\textbf {W}}}$ follows a logistic regression model, $\text{ logit } P(\text {X} = 1 \vert {{\textbf {W}}}) = \eta _0 + \varvec{\eta }_1^T{{\textbf {W}}}$, where $\eta _0$ is the intercept and $\varvec{\eta }_1$ is the logistic regression coefficient for **W**. The first adjustment for left truncation is using the modified definition of the risk set, i.e., replacing $Y_i(t)$ with $Y^*_i(t)$ in the Cox model. In addition, we consider the imputation model $p(\text {X} \vert T, \delta , L, {{\textbf {W}}}, T > L)$ in Equation (2).

2 $$\begin{aligned} \begin{aligned} p(\text {X} \vert {{\textbf {W}}}, T, \delta , L, T>L)&\propto f(T,\delta \vert \text {X}, {{\textbf {W}}})p(\text {X}\vert {{\textbf {W}}}). \end{aligned} \end{aligned}$$

The imputation model has two components: $p(\text {X} \vert {{\textbf {W}}})$ is the user-specified conditional distribution of the missing data X under no truncation, while the Cox model implies that $f(T,\delta \vert \text {X}, {{\textbf {W}}}) = h(T \vert \text {X},{{\textbf {W}}})^\delta S(T \vert \text {X},{{\textbf {W}}})$. In the supplementary materials, we provide further details on how Equation (2) is derived. This involves two key independence assumptions on the truncation time *L*. The first is the previously mentioned standard assumption for Cox regression with left-truncated data $U \perp (L,C) \vert \text {X}, {{\textbf {W}}}, T>L$, which allows for unbiased estimation of the Cox model parameters through the risk set adjustment. If the second independence assumption $\text {X} \perp (L,C) \vert {{\textbf {W}}}$ also holds, then $p(\text {X} \vert {{\textbf {W}}}, T, \delta , L, T>L)$ reduces to the imputation model that is appropriate for non-truncated data $p(\text {X} \vert {{\textbf {W}}}, T, \delta )$, which is proportional to the right side of Equation (2).

Although the target for the missing data component is $p(\text {X} \vert {{\textbf {W}}})$, the observed data come from the selection-biased density $p(\text {X} \vert {{\textbf {W}}},T>L)$. Depending on the severity of selection bias, the population covariate distribution may be modeled poorly with a biased sample, causing the imputation model to be incorrectly estimated. As such, MI will perform well when applied to truncated data when X and *L* are conditionally independent and the population and sample covariate distributions are similar. When $p(\text {X} \vert {{\textbf {W}}},T>L)$ cannot approximate $p(\text {X} \vert {{\textbf {W}}})$, MI will be biased due to the effect of selection bias on the sample covariate distribution.

It is important to note that $H_0(T)$ is unknown. White and Royston [25] recommend estimating this quantity with the nonparametric Nelson-Aalen estimator [1, 12] of the cumulative hazard, *H*(*T*). This works well when the hazard ratios are small, such that $H_0(T) \approx H(T) = H_0(T)\exp (\beta _1 \text {X}+\varvec{\beta }_2^T {{\textbf {W}}})$. Since we are working with all categorical covariates, the approximation could be improved by stratifying on different covariate combinations if the stratum-specific sample sizes are sufficiently large.

### Augmented Inverse Probability Weighting

IPW and MI require that the user correctly specifies a model related to the missing data (i.e., the reason for missingness or the missing data itself). The AIPW framework, first proposed by Robins et al. [16], offers some robustness against model misspecification and potential efficiency gains over IPW. AIPW has been considered in Cox regression analysis under right censoring in the absence of left truncation [14, 23, 26]. We modify the AIPW score equation developed under right censoring only to account for left truncation by a risk set adjustment, i.e., replacing $Y_i(t)$ with $Y_i^*(t)$. The AIPW score equation is given in Equation (3).

3 $$\begin{aligned} \begin{aligned} \frac{1}{n}\sum _{i=1}^n\left\{ \frac{R_i \delta _i}{\pi _i}\bigg [ \begin{pmatrix} \text {X}_i \\ {{\textbf {W}}}_i \end{pmatrix} - \frac{S_{AW}^{(1)}(\varvec{\beta }, T_i)}{S_{AW}^{(0)}(\varvec{\beta }, T_i)} \bigg ] + A_i(\varvec{\beta }) \right\} = {\varvec{0}}, \end{aligned} \end{aligned}$$

where

$$\begin{aligned} \begin{aligned} r_i^{(0)}(\varvec{\beta })&= \exp (\beta _1\text {X}_i + \varvec{\beta }_2^T{{\textbf {W}}}_i),\quad r_i^{(1)} (\varvec{\beta }) = (\text {X}_i, {{\textbf {W}}}_i^T)^T \exp (\beta _1\text {X}_i + \varvec{\beta }_2^T{{\textbf {W}}}_i),\\ S^{(m)}_{AW}(\varvec{\beta }, t)&= \frac{1}{n}\sum _{i=1}^n \bigg [\frac{R_i}{\pi _i} Y_i^*(t) r_i^{(m)}(\varvec{\beta }) + \left( 1-\frac{R_i}{\pi _i}\right) Y_i^*(t)E(r_i^{(m)} \vert T_i, \delta _i, {{\textbf {W}}}_i)\bigg ] \text{ for } m = 0,1,\\ A_i(\varvec{\beta })&=\left( 1-\frac{R_i}{\pi _i}\right) \int \bigg \{ E\bigg [\begin{pmatrix}\text {X}_i\\ {{\textbf {W}}}_i \end{pmatrix} dN_i(u)\vert T_i, \delta _i, {{\textbf {W}}}_i\bigg ]\\&\quad -\frac{S^{(1)}_{AW}(\varvec{\beta }, T_i)}{S^{(0)}_{AW} (\varvec{\beta }, T_i)}E(dN_i(u)\vert T_i, \delta _i, {{\textbf {W}}}_i) \bigg \}. \end{aligned} \end{aligned}$$

The conditional expectations in the augmentation term $A_i(\varvec{\beta })$ rely on estimating $H_0(t)$ and $p(\text {X} \vert {{\textbf {W}}})$. Estimation of the cumulative baseline hazard is accomplished through a Breslow-type estimator, ${\hat{H}}_0(t) = \frac{1}{n} \sum _{i=1}^n I(T_i\le t)\delta _i/S^{(0)}_{AW}(\varvec{\beta }, T_i)$. $p(\text {X} \vert {{\textbf {W}}})$ is specified by the user. Let $\varvec{\eta }$ be the parameters governing $p(\text {X} \vert {{\textbf {W}}})$ and $\varvec{\psi _{\eta }}$ be the score function for that model. Estimation of $\varvec{\eta }$ is performed using the score equation given in Eq (4).

4 $$\begin{aligned} \begin{aligned} \frac{1}{n} \sum _{i=1}^n \left\{ \frac{R_i}{\pi _{i}}\varvec{\psi _{\eta }} (\text {X}_i, {{\textbf {W}}}_i) + \bigg (1-\frac{R_i}{\pi _{i}}\bigg )E \bigg [\varvec{\psi _{\eta }}(\text {X}_i, {{\textbf {W}}}_i) \vert T_i,\delta _i,{{\textbf {W}}}_i\bigg ]\right\} = {\varvec{0}}. \end{aligned} \end{aligned}$$

AIPW estimates are unbiased if either $\pi$ or $p(\text {X} \vert {{\textbf {W}}}$) is correctly specified (the so-called double robustness property), making it a more flexible approach than other methods. When the data are MAR and $\pi$ are misspecified, AIPW will correct the bias of IPW so long as $p(\text {X} \vert {{\textbf {W}}})$ is correctly specified and consistently estimated. Under MCAR (or MAR with correct $\pi$), AIPW may also offer increased efficiency over standard weighting methods [18].

We consider the score equation given in Equation (3) utilizing the imputation model derived in Sect. 2.3, $p(\text {X} \vert {{\textbf {W}}},T,\delta ,L,T>L)$. We estimate the standard error using bootstrap resampling with 500 replicated datasets. Similar to MI, the proposed imputation model requires that $\text {X} \perp L \vert {{\textbf {W}}}$ and that the parameters governing $p(\text {X} \vert {{\textbf {W}}})$ can be estimated well with the biased sample. In cases of extreme selection bias, the missing data model cannot be estimated correctly, so consistency of AIPW relies on the correct specification of $\pi$.

## Simulation Study

Through simulation studies, we aim to quantify the effect of truncation on missing data methods, particularly those involving modeling the missing covariate distribution. Simulation parameters were selected to mimic our motivating data example. We also varied these parameters to explore a variety of settings. We considered a sample size of n = 300, with results for n = 500 presented in the supplementary materials. We began by generating data for $\frac{n}{(1-q)}$ observations, where *q* is the truncation rate. Study entry times, *L*, were generated from a $c_1$Beta(6,1.5) distribution, where $c_1$ was varied according to the truncation rate. We considered truncation rates of 0, 0.15, 0.25, 0.5, and 0.75. Binary covariates ${{\textbf {W}}} = (W_1, W_2, W_3)^T$ were generated from independent Bernoulli(0.5) distributions. The primary covariate of interest, X, was generated according to a logistic regression model with mean $\eta _0 + \varvec{\eta }_1^T{{\textbf {W}}}$, where $\eta _0 = 0.5$ and $\varvec{\eta }_1 = (\log (2), -\log (2),\log (1.5))^T$.

*U* followed a Cox proportional hazards model with hazard $h(t \vert \text {X},{{\textbf {W}}}) = h_0(t)\exp (\beta _1\text {X} + \varvec{\beta }^T_2{{\textbf {W}}})$, where $h_0(t) = \rho \kappa (\rho t)^{\kappa -1},\ \rho =0.1,\ \kappa =0.5,\ \varvec{\beta }^T_2 = (\log (2), -\log (2), \log (1.5))$. $\beta _1$ values of both $\log (2)$ and $\log (4)$ were tested. Subjects with $L_i > T_i$ were removed from the sample to produce a final sample of approximately size *n*. Finally, random censoring times were introduced. $C_i'$ was generated from a Weibull(4, $c_2$), where $c_2$ was chosen to produce the desired censoring rate. The censoring time was $C_i = c_1 + C_i'$, so that $P(C > L) = 1$. Censoring rates of $\lambda$ = 0.1 and 0.5 were considered.

The final step of data generation occurred with introducing missingness in the covariate X. First, we implemented an MCAR mechanism in which $\pi _i$ was constant across subjects. We tested missing data rates of 0.25 and 0.5. Under MCAR, $\pi$ can be trivially estimated by the sample proportion of observed data. As a result, misspecification of $\pi$ is unlikely in practice. Therefore, we studied IPW and AIPW with correctly specified $\pi$ model, a logistic regression model with outcome *R* and predictor $W_1$. We also tested CC, MI, and the complete data (CD) estimate, which applied the Cox model to the dataset with no missing values. For MI, 100 imputed data-sets were generated and $H_0(T)$ was estimated using the Nelson–Aalen estimator for *H*(*T*). The standard error for AIPW was computed using bootstrap sampling with 500 bootstrap replicates.

**Fig. 2:**
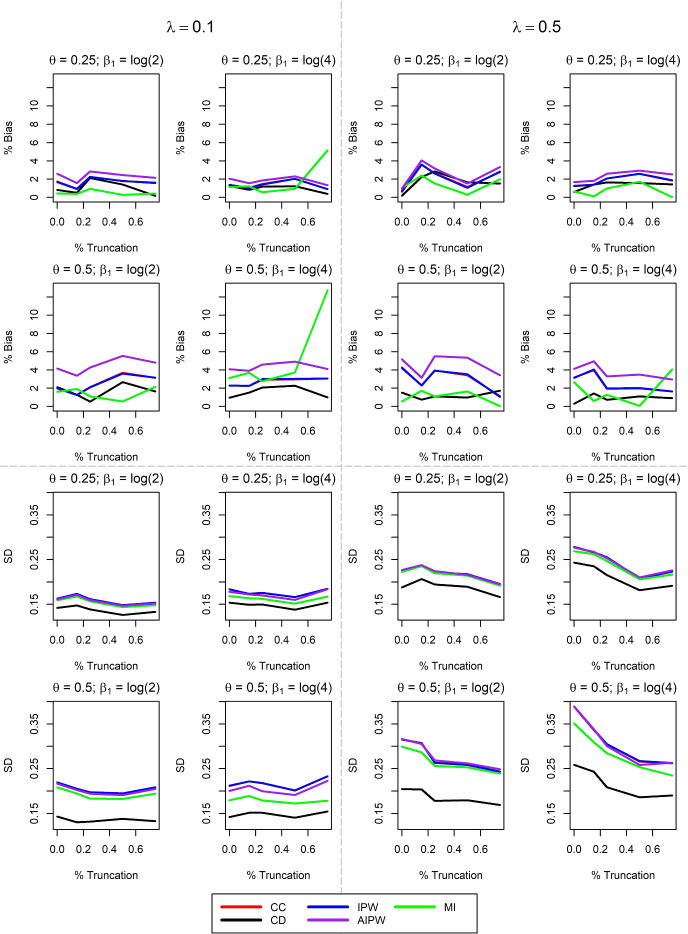
Extended Simulation Results for MCAR Missingness. The sample size is $n=300$, $\beta _1$ is the log-hazard ratio of the missing covariate X, $\lambda$ is the censoring rate, and $\theta$ is the proportion of data with X missing

Simulation results for the MCAR mechanism under 10% censoring, 50% missing data, and $\beta _1 = \log (4)$ are shown in Table 1. As expected, CC and IPW are unbiased and have similar variability regardless of the degree of truncation. AIPW also has no bias, which can be attributed to the correctly specified $\pi$ model. Under low to moderate truncation, MI is unbiased. The only method exhibiting bias greater than 10% is MI under 75% truncation due to the departure of the sample covariate distribution from the target population covariate distribution. The MI standard error estimator also performs worse as truncation increases, which can occur with a misspecified imputation model [8]. Figure 2 demonstrates the effect of altering $\beta _1$ as well as missingness and censoring rates. $\theta$ refers to the proportion of subjects for whom X is missing, and $\lambda$ represents the proportion of subjects whose event time is censored. Similar to Table 1, CC and IPW are unbiased across all truncation, missingness, and censoring percentages. These methods also have similar standard deviations because ${\hat{\pi }}$ is approximately constant under MCAR. AIPW is also unbiased with increasing truncation, due to the fact that $\pi$ is correctly specified. Under many circumstances, AIPW has similar efficiency to CC and IPW. When AIPW has an efficiency gain, this advantage decreases with moderate or large truncation. In many settings, there is an increase in bias of MI as truncation increases. In these simulations, bias is most extreme with larger $\beta _1$ and lower censoring. MI often has smaller variance than other missing data methods, though it is important to note that small MI variance may be accompanied by larger MI bias. Very similar results were obtained using a larger sample size (n=500), which are presented in Figure S.1 in the supplementary information.

**Table 1:** Simulation results with sample size $n=300$, X regression coefficient $\beta _1 = \log (4)$, 10% censoring, and 50% MCAR missingness. The nominal confidence interval coverage probability is 95%

$\gamma$ $^{1}$	Value	CC	CD	IPW	AIPW	MI
0	Bias	0.032	0.018	0.032	0.059	$-$0.043
	% Bias	2.326	1.330	2.284	4.236	$-$3.098
	SD	0.227	0.155	0.227	0.215	0.193
	mean(SE)	0.224	0.155	0.220	0.225	0.223
	Cov. Prob	0.956	0.950	0.948	0.952	0.980
0.15	Bias	0.029	0.014	0.029	0.051	$-$0.053
	% Bias	2.124	0.997	2.118	3.670	$-$3.842
	SD	0.236	0.160	0.236	0.224	0.200
	mean(SE)	0.215	0.149	0.211	0.215	0.221
	Cov. Prob	0.928	0.926	0.920	0.922	0.954
0.25	Bias	0.037	0.022	0.037	0.064	$-$0.040
	% Bias	2.670	1.572	2.692	4.587	$-$2.854
	SD	0.203	0.145	0.204	0.192	0.174
	mean(SE)	0.210	0.146	0.205	0.208	0.219
	Cov. Prob	0.950	0.942	0.944	0.950	0.988
0.5	Bias	0.053	0.020	0.054	0.072	$-$0.047
	% Bias	3.849	1.437	3.864	5.216	$-$3.390
	SD	0.199	0.135	0.199	0.190	0.169
	mean(SE)	0.202	0.140	0.195	0.200	0.217
	Cov. Prob	0.948	0.954	0.936	0.950	0.988
0.75	Bias	0.037	0.016	0.038	0.053	$-$0.176
	% Bias	2.698	1.137	2.727	3.842	$-$12.716
	SD	0.223	0.149	0.223	0.218	0.181
	mean(SE)	0.225	0.156	0.215	0.224	0.270
	Cov. Prob	0.946	0.958	0.938	0.946	0.976

$^1$ $\gamma$ is the simulated truncation rate

Next, we compared these methods under an MAR mechanism in which *R* was related to the censoring indicator, $\delta$. *R* was generated according to a logistic regression model with mean $a + \log (4)\delta$, where *a* was varied across parameter settings to consistently produce the desired missing data rate. Under the MAR mechanism, we compared IPW and AIPW with both correct and incorrect $\pi$ models. When $\pi$ was correctly specified, it was a logistic regression model with $\delta$ as the only predictor. When it was incorrectly specified, $W_1$, $W_2$, and $W_3$ were used to predict missingness. We denote methods using a correctly specified $\pi$ model as IPW($\pi _C$) and AIPW($\pi _C$). Under incorrectly specified $\pi$ model, these methods are referred to as IPW($\pi _I$) and AIPW($\pi _I$).

Simulation results for MAR data are presented in Table 2. Due to the MAR mechanism, CC is biased, though under the selected settings, this bias decreases as truncation increases. IPW addresses this bias well so long as $\pi$ correctly specified, regardless of percent truncation (illustrated by comparing the two IPW columns in Table 2). AIPW with correct $\pi$ is also unbiased across all levels of truncation tested (though the bias reduction at high levels of truncation is low due to smaller CC bias). Notably, AIPW is more efficient than IPW when $\pi$ is correct and the data are not truncated, and this efficiency gain disappears with larger truncation. AIPW is expected to be more efficient than IPW when both the missingness and imputation models are correctly specified and consistently estimated. This is the case in our simulations with zero truncation, and we do see that the SD of AIPW$(\pi _C)$ is consistently smaller than that of IPW for those settings, albeit by a small amount $<10\%$. The degree of expected efficiency gain depends on the distribution of the data, and happens to be small for the settings considered in this paper. Thus, our simulation results for AIPW$(\pi _C)$ are consistent with general expectations. When left truncation is present, the covariate distribution in the imputation model is not estimated consistently due to the biased sample, so we cannot necessarily expect AIPW$(\pi _C)$ to have an efficiency advantage over IPW. AIPW with $\pi$ misspecified can effectively reduce bias when compared to CC with little or no truncation, but they perform similarly at high truncation rates. MI is unbiased with low truncation percentages and can become severely biased with more extreme selection bias.

**Fig. 3:**
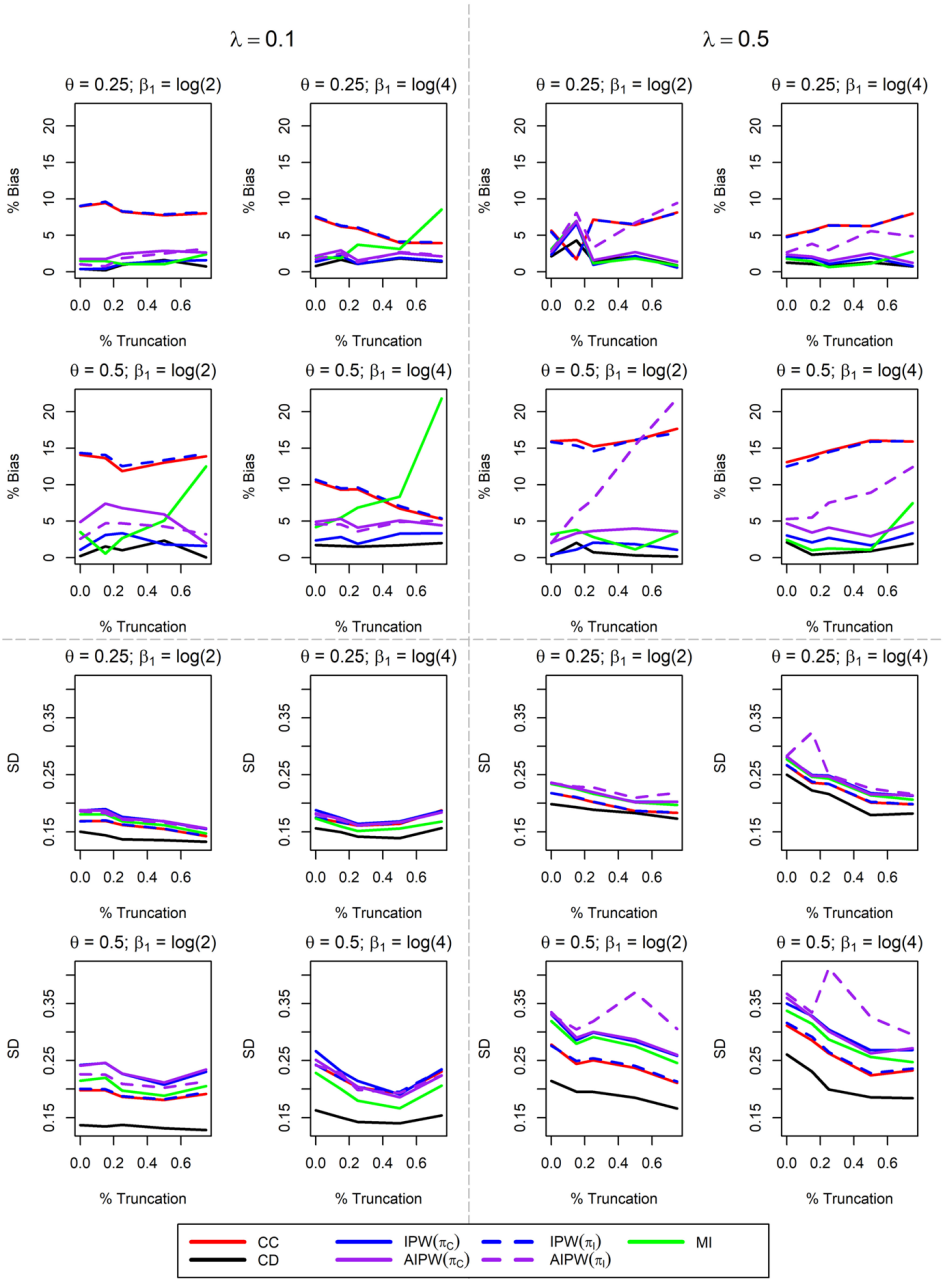
Extended Simulation Results for $\delta$-dependent MAR Missingness. The sample size is $n=300$, $\beta _1$ is the log-hazard ratio of the missing covariate X, $\lambda$ is the censoring rate and $\theta$ is the proportion of data with X missing

Figure 3 explores the impact of altering various parameters of the $\delta$-dependent MAR simulations. CC is biased across most settings and truncation rates. IPW and AIPW successfully eliminate this bias when $\pi$ is correctly specified, and good performance does not rely on truncation percentage. When $\pi$ is misspecified, however, the relationship between the truncation proportion and the bias and variance of AIPW($\pi _I$) under different truncation scenarios is complex and not necessarily monotone. Since both the missingness model and the imputation model are not estimated consistently when the truncation proportion is significant, the expected behavior of AIPW($\pi _I$) is unknown. MI and AIPW($\pi _I$) can become extremely biased when truncation is substantial. Notably, the performance of these methods relies on multiple factors, such as censoring rate, missingness rate, and hazard ratio of the missing variable. As such, predicting poor performance of MI and AIPW($\pi _I$) can be difficult due to the complex nature of these data. These methods often have similar variabilities, with MI often having an efficiency gain with smaller truncation rates. $\delta$-dependent MAR simulation results with a sample size of 500 are provided in Supplemental Figure S.2. Many of the same trends persist with larger sample size, and MI and AIPW($\pi _I$) remain biased under some settings with high truncation. However, increasing *n* slightly decreases MI bias, while AIPW($\pi _I$) bias is comparable between sample sizes.

**Table 2:** Simulation results with sample size $n=300$, X regression coefficient $\beta _1 = \log (4)$, 10% censoring and 50% $\delta$-dependent MAR missingness. The nominal confidence interval coverage probability is 95%

$\gamma$ $^1$	Value	CC	CD	Correct $\pi$		Incorrect $\pi$		MI
				IPW	AIPW	IPW	AIPW	
0	Bias	$-$0.141	0.018	0.035	0.074	$-$0.146	0.063	$-$0.053
	% Bias	$-$10.184	1.314	2.535	5.307	$-$10.549	4.562	$-$3.844
	SD	0.221	0.157	0.246	0.230	0.223	0.219	0.212
	mean(SE)	0.216	0.155	0.243	0.232	0.214	0.232	0.267
	Cov. Prob	0.900	0.952	0.940	0.954	0.894	0.900	0.982
0.15	Bias	$-$0.138	0.024	0.035	0.066	$-$0.141	0.056	$-$0.078
	% Bias	$-$9.953	1.701	2.499	4.734	$-$10.204	4.011	$-$5.658
	SD	0.222	0.154	0.241	0.231	0.223	0.225	0.204
	mean(SE)	0.207	0.149	0.229	0.220	0.204	0.220	0.277
	Cov. Prob	0.870	0.942	0.924	0.930	0.856	0.870	0.984
0.25	Bias	$-$0.130	0.012	0.013	0.047	$-$0.135	0.041	$-$0.113
	% Bias	$-$9.394	0.894	0.960	3.373	$-$9.726	2.979	$-$8.142
	SD	0.204	0.137	0.219	0.203	0.206	0.201	0.187
	mean(SE)	0.201	0.145	0.218	0.210	0.198	0.211	0.278
	Cov. Prob	0.888	0.960	0.942	0.950	0.882	0.888	0.990
0.5	Bias	$-$0.084	0.022	0.051	0.078	$-$0.089	0.075	$-$0.115
	% Bias	$-$6.078	1.572	3.708	5.596	$-$6.388	5.446	$-$8.265
	SD	0.202	0.150	0.209	0.202	0.203	0.205	0.177
	mean(SE)	0.196	0.140	0.206	0.198	0.192	0.198	0.267
	Cov. Prob	0.936	0.928	0.930	0.914	0.918	0.936	0.996
0.75	Bias	$-$0.075	0.024	0.045	0.064	$-$0.076	0.072	$-$0.304
	% Bias	$-$5.400	1.747	3.279	4.644	$-$5.476	5.195	$-$21.904
	SD	0.230	0.164	0.233	0.229	0.229	0.230	0.205
	mean(SE)	0.220	0.156	0.222	0.220	0.211	0.221	0.337
	Cov. Prob	0.930	0.928	0.924	0.932	0.920	0.930	0.956

$^1$ $\gamma$ is the simulated truncation rate

Finally, we considered the case where *R* was related to the observed time, *T*. The true underlying missing data mechanism, $P(R=1 \vert T)$, was a logistic regression model with mean $\alpha _0 - \alpha _1\sqrt{T}$. Because the distribution of *T* changes with the censoring rate, we chose $\alpha _1 = \log (1.25)$ under 10% censoring and $\alpha _1 = \log (3)$ under 50% censoring. $\alpha _0$ was varied across parameter settings to consistently produce the specified amount of missing data. Similar to the $\delta$-dependent MAR case, the correctly specified $\pi$ model used only $\sqrt{T}$ to predict *R*. The incorrect $\pi$ model had predictors $W_1$, $W_2$, and $W_3$.

**Fig. 4:**
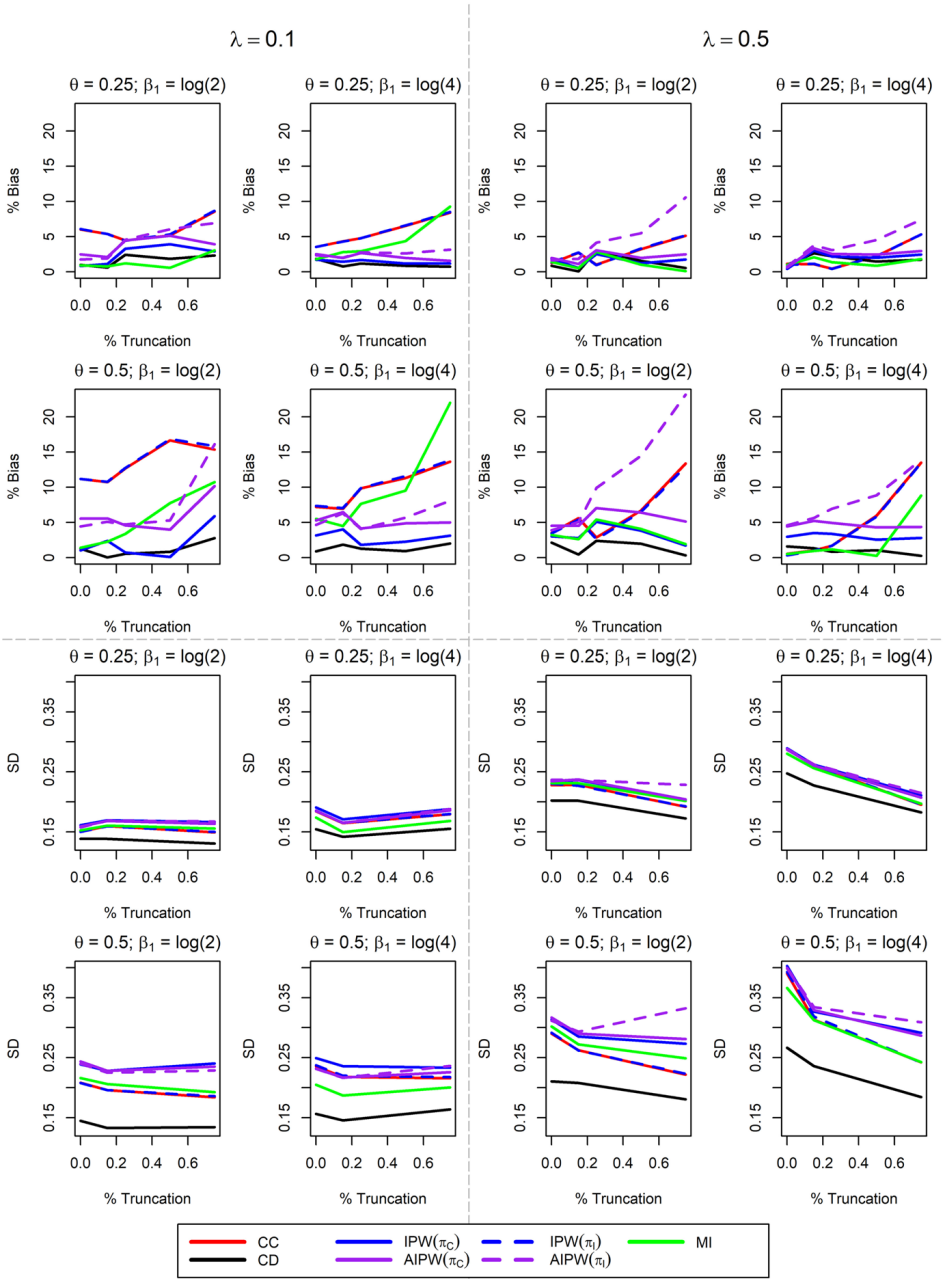
Extended Simulation Results for *T*-dependent MAR Missingness. The sample size is $n=300$, $\beta _1$ is the log-hazard ratio of the missing covariate X, $\lambda$ is the censoring rate, and $\theta$ is the proportion of data with X missing

Results for the case with 10% censoring, 50% missingness, and $\beta _1 = \log (4)$ are provided in Table 3. Similar to the $\delta$-dependent MAR case, CC is biased, though in this scenario, bias increases with larger truncation rates. IPW with $\pi$ incorrect is similarly biased. IPW and AIPW both reduce bias compared to CC when $\pi$ is correctly specified. AIPW with $\pi$ incorrect and MI both have bias exceeding 10% when truncation is extreme. Figure 4 displays additional simulation results altering the amount of censoring, rate of missing data, and hazard ratio. Again, MI and AIPW($\pi _I$) are often biased with larger truncation rates. However, hazard ratio, censoring, and missingness rates all impact the effect of increasing truncation on the performance of these methods. Results for the larger sample size are presented in Supplemental Figure S.3. As previously noted, MI bias is slightly mitigated with larger sample size, though still substantial in many cases.

**Table 3:** Simulation results with sample size $n=300$, X regression coefficient $\beta _1 = \log (4)$, 10% censoring, and 50% *T*-dependent MAR missingness. The nominal confidence interval coverage probability is 95%

$\gamma$ $^1$	Value	CC	CD	Correct $\pi$		Incorrect $\pi$		MI
				IPW	AIPW	IPW	AIPW	
0	Bias	$-$0.098	0.018	0.042	0.072	$-$0.100	0.063	$-$0.071
	% Bias	$-$7.086	1.312	3.058	5.159	$-$7.212	4.570	$-$5.097
	SD	0.229	0.148	0.249	0.228	0.231	0.226	0.204
	mean(SE)	0.223	0.155	0.236	0.235	0.221	0.236	0.248
	Cov. Prob	0.920	0.958	0.922	0.934	0.914	0.920	0.970
0.15	Bias	$-$0.116	0.017	0.043	0.080	$-$0.118	0.073	$-$0.086
	% Bias	$-$8.355	1.194	3.133	5.736	$-$8.484	5.248	$-$6.236
	SD	0.216	0.144	0.237	0.219	0.217	0.219	0.191
	mean(SE)	0.212	0.149	0.226	0.222	0.209	0.223	0.256
	Cov. Prob	0.918	0.966	0.936	0.932	0.898	0.918	0.986
0.25	Bias	$-$0.140	0.019	0.026	0.059	$-$0.140	0.059	$-$0.114
	% Bias	$-$10.075	1.364	1.890	4.282	$-$10.112	4.274	$-$8.253
	SD	0.211	0.148	0.230	0.215	0.213	0.218	0.184
	mean(SE)	0.206	0.145	0.219	0.215	0.201	0.215	0.258
	Cov. Prob	0.896	0.950	0.936	0.936	0.886	0.896	0.984
0.5	Bias	$-$0.154	0.022	0.037	0.071	$-$0.157	0.083	$-$0.132
	% Bias	$-$11.085	1.606	2.681	5.106	$-$11.328	5.990	$-$9.557
	SD	0.209	0.150	0.230	0.224	0.212	0.232	0.194
	mean(SE)	0.195	0.140	0.209	0.203	0.190	0.203	0.249
	Cov. Prob	0.864	0.920	0.916	0.898	0.850	0.864	0.966
0.75	Bias	$-$0.183	0.035	0.078	0.098	$-$0.187	0.141	$-$0.325
	% Bias	$-$13.218	2.527	5.626	7.081	$-$13.455	10.192	$-$23.432
	SD	0.219	0.153	0.242	0.225	0.221	0.237	0.202
	mean(SE)	0.211	0.156	0.226	0.216	0.202	0.216	0.331
	Cov. Prob	0.858	0.948	0.916	0.898	0.818	0.858	0.948

$^1$ $\gamma$ is the simulated truncation rate

## Application to Parkinson’s Disease Cognition Research

We explore the performance of missing data methods when applied to a study evaluating associations between cerebrospinal fluid biomarkers and cognitive decline in PD patients. The study cohort was followed up by the University of Pennsylvania Parkinson’s Disease Research Center (PDRC). Cognitively normal PD patients were enrolled in the study. PD diagnosis (time 0) occurred prior to study entry for all participants, producing a left-truncated sample. Participants were followed until they developed the event of interest, defined as mild cognitive impairment (MCI) or dementia. The dataset contains 223 participants, of which 108 (48%) were censored. We use the method presented by Wang [24] to quantify the proportion of subjects in the population who experienced the outcome prior to study entry, which is estimated to be 45% for this dataset. In other words, the estimated left truncation rate is 0.45.

We aim to use Cox regression to evaluate the association between abnormal CSF $A\beta _{42}$ on cognitive decline in PD, controlling for age at study entry and baseline cognitive score, measured through the Dementia Rating Scale (DRS). Abnormal CSF $A\beta _{42}$ is a binary variable defined as a concentration of CSF $A\beta _{42} \le$ 192 pg/ml [20]. Obtaining a CSF sample is expensive and invasive for the patient. As such, information on this variable was obtained for only a subset of the cohort. Further, CSF samples taken more than 8 months after study enrollment are considered not representative of baseline values and treated as missing. 133 subjects (60% of total cohort) did not have CSF $A\beta _{42}$ information available. Among the complete cases, 12 (13%) had an abnormal concentration of CSF $A\beta _{42}$. We employ categorical versions of the age (younger vs. older) and DRS (lower vs. higher score) variables. The young group are all subjects with an age at baseline less than 67 years and the low DRS group are subjects with a baseline DRS score less than 139. These thresholds are selected because they are the median observed values in the sample.

AIPW and MI require a model for the conditional distribution of abnormal CSF $A\beta _{42}$ to perform AIPW and MI. Since abnormal CSF $A\beta _{42}$ is binary, we specify this distribution using a logistic regression model. At baseline, subjects undergo genetic screening for the APOE $\epsilon _4$ allele. Previous studies have demonstrated that presence of this allele is associated with decreased CSF $A\beta _{42}$ [22]. As such, APOE $\epsilon _4$ information is an important explanatory variable in modeling missing CSF $A\beta _{42}$ values. We also include binary age and binary DRS score as predictors of abnormal CSF $A\beta _{42}$. The MI imputation model uses the Nelson-Aalen estimate of *H*(*T*) and appropriate interaction terms as described by White and Royston [25]. We generate 100 imputed datasets. In AIPW, 500 bootstrap replicates are used to estimate the standard error.

To perform IPW and AIPW, we must specify a model to estimate the probability of observing CSF $A\beta _{42}$ at baseline ($\pi$). We use a logistic regression model with outcome *R* to estimate $\pi$. Model misspecification may occur when important information is left out of the model. Thus, we include all fully observed variables as predictors in this model. Binary *L* is defined as early vs. late entry, where subjects who enter late are those who came under observation more than 1562 days after diagnosis. Similarly, binary *T* is defined as early vs late event time, where late events occurred more than 3391 days after diagnosis. Again, these thresholds are selected because they are the median observed values. As a result, the final $\pi$ model contains predictors $\delta$, APOE $\epsilon _4$, older age, higher DRS score, late *L*, and late *T*. To perform IPW and AIPW, $\pi$ is estimated using the fitted values of this model. We also use this model to assess possible missing data mechanisms for this data. Using Wald tests and nested likelihood ratio tests, we conclude that the only important predictors of missingness are age (p = 0.047) and DRS score (p = 0.001). Thus, the $\pi$ model suggests that missingness may be MCAR in this dataset.

**Table 4:** Penn PDRC Data Analysis: Regression coefficient estimates for abnormal CSF $A\beta _{42}$

Method	${\hat{\beta }}$	Std. Error	p-value
CC	0.972	0.421	0.02
IPW	0.952	0.428	0.03
AIPW	1.065	0.402	0.01
MI	0.650	0.328	0.05

Table 4 presents regression coefficient estimates for abnormal CSF $A\beta _{42}$ obtained using a variety of missing data techniques. Using complete cases only, abnormal CSF $A\beta _{42}$ is significantly associated with increased risk of MCI or dementia (${\hat{\beta }}_{\text {CC}}$ = 0.972, p = 0.02). Results using IPW are very similar to CC (${\hat{\beta }}_{\text {IPW}}$= 0.952, p = 0.03). The point estimate of AIPW is comparable to CC and IPW (${\hat{\beta }}_{\text {AIPW}}$= 1.050). Under the assumed MCAR mechanism, it is expected that CC, IPW, and AIPW produce similar estimates, since $\pi$ is usually correctly estimated under MCAR. MI results in a much smaller effect estimate and a p-value equal to the threshold for significance (${\hat{\beta }}_{\text {MI}}$= 0.650, p = 0.05). In addition, its standard error is smaller than the other three methods, due in part to the smaller magnitude of its coefficient estimate. The large difference in estimates obtained may be due to violation of either assumption made by the imputation model. The first assumption is that the missing covariate and entry times are independent conditional on other covariates. In this study, CSF $A\beta _{42}$ value is not known prior to study entry. As such, it seems unlikely that the CSF $A\beta _{42}$ value could influence the time from PD diagnosis to study entry, except perhaps through the influence of related risk factors. While the conditional independence assumption seems plausible, the estimated amount of truncation is moderate. In this case, it is plausible that the sample covariate distribution is not representative of the population. This would cause bias in estimation of the imputation model. Our scientific conclusion based on the above analyses is that abnormal CSF $A\beta _{42}$ is related to increased risk of conversion to MCI or dementia among PD patients.

## Discussion

This study investigated how commonly used approaches for handling missing covariate data in the Cox model perform when the data are left-truncated. In particular, the biased sample complicates estimating the missing covariate distribution, even when it is modeled correctly (i.e., the parametric structure is correctly specified). In many practical settings, the estimated missing data model may be approximately unbiased, especially if truncation is very low. When the truncation scenario is more extreme, the biased covariate distribution can cause poor MI performance. AIPW may rely more heavily on correct estimation of the probability of having non-missing covariates when the data is MAR. Notably, poor performance of these methods relies on many factors and can be difficult to predict. This study motivates future research surrounding unbiased estimation of covariate distribution parameters under left truncation in the presence of missing data. Such a method could ensure unbiased results under many truncation scenarios, making it an attractive missing data method for left-truncated survival data.

We considered only the case where the missing covariate is binary, observed covariates are categorical, and the two are related through a logistic regression model. In this situation, it can be shown that the correct imputation model is also a logistic regression model. With other data types, the correct imputation model does not follow a standard distribution. Approximations to the true imputation model have been studied in the absence of left truncation [25]. As such, when truncation is introduced, it would be unclear whether observed bias could be attributed to poor approximation of the imputation model (due to continuous data types) or improper estimation of the imputation model parameters (because the sample covariate distribution is biased). Bartlett et al. [3] propose an MI procedure that can overcome this limitation of standard MI by generating random draws from nonstandard distributions. Another option for fitting the imputation model is through a nonparametric method such as random forests. Exploring the bias pattern of these methods in the context of left truncation would be an interesting future research direction.

Our simulation results demonstrate that predicting the performance of methods that require modeling the missing covariate distribution is complex. Regardless of other parameters, these models perform well when the amount of truncation is very small, perhaps 15% or less. In these cases, MI and AIPW will likely perform as expected under right censoring only, since the sample covariate distribution approximates the population distribution well. We recommend using MI and AIPW when truncation is very low due to increased efficiency over IPW.

When truncation is moderate or substantial, careful consideration is required to select an appropriate missing data method. When MCAR can be assumed, an investigator may choose CC, IPW, or AIPW to ensure unbiased results (since $\pi$ is usually correctly specified under MCAR). Under MAR, there is no guarantee that any method will perform well. In particular, because the survival outcome is related to both the selection bias and missing data mechanism, altering parameters can have complex consequences on model performance. As an example, in our simulations, AIPW($\pi _I$) was unbiased under $\delta$-dependent missingness, high truncation, and low censoring (Table 2), which is unexpected due to misspecification of both models. However, the censoring rate was very low, so omitting $\delta$ from the $\pi$ model did not have a large impact on estimation. Thus, AIPW($\pi _C$) and AIPW($\pi _I$) performed similarly in this specific scenario.

Due to the complexity of these data under MAR, selecting the appropriate method to address missing covariate data may be challenging. In general, CC is biased due to the non-representative complete case subsample. IPW and AIPW both require correct specification of the probability of missingness model. Practical guidelines for specifying this model are provided by Seaman and White [19]. Unless truncation is very small, MI modeling may also be biased even when the imputation model is correctly specified. When the probability of missingness depends on the survival outcome, we recommend using a variety of missing data methods and critically evaluating the assumptions of each.

## Supplementary information

The online version of this article contains supplementary material available at the journal’s website. R code for CC, IPW, MI, and AIPW are provided in the supplementary materials.

## Supplementary Information

Below is the link to the electronic supplementary material.

Supplementary file 1 (pdf 1979 KB)
