# Supplementary material for: Analyzing Left-Truncated Samples with the Cox Model in the Presence of Missing Covariates
Source: Stat Biosci. 2024 Jul 2;17(2):555–74. doi: 10.1007/s12561-024-09442-9 (PMC12122580; doi:10.1007/s12561-024-09442-9)
Supplement: Supplementary file 1 — (pdf 1979 KB) [file 12561_2024_9442_MOESM1_ESM.pdf]

Supplemental Materials for Analyzing Left-Truncated Samples with the Cox Model in the Presence of Missing Covariates

Omar Vazquez, Hayley M. Locke, & Sharon X. Xie

Department of Biostatistics, Epidemiology, and Informatics, University of Pennsylvania
Perelman School of Medicine, Philadelphia, PA, USA.

1 Derivation of Equation (2) in the main text

First, we relate the joint distribution of all the observed variables after truncation to their pre-truncation distribution:

$$f(T, \delta, L, \mathbf{W}, \mathrm{X}, T > L) = I(L < T) f(T, \delta, L, \mathbf{W}, \mathrm{X}) / P(L < T).$$

Marginalizing over the covariate X, we have

$$f(T, \delta, L, \mathbf{W}, T > L) = I(L < T) f(T, \delta, L, \mathbf{W}) / P(L < T).$$

Then the conditional distribution for the imputation model is

$$\begin{aligned}
p(\mathrm{X}|\mathbf{W}, T, \delta, L, T > L) &= f(T, \delta, L, \mathbf{W}, \mathrm{X}, T > L) / f(T, \delta, L, \mathbf{W}, T > L) \\
&= f(T, \delta, L, \mathbf{W}, \mathrm{X}) / f(T, \delta, L, \mathbf{W}) \\
&= p(\mathrm{X}|\mathbf{W}, T, \delta, L) \\
&\propto f(T, \delta|\mathrm{X}, \mathbf{W}, L) p(\mathrm{X}|\mathbf{W}, L) \qquad \text{(dropping constants in X)} \\
&= f(T, \delta|\mathrm{X}, \mathbf{W}) p(\mathrm{X}|\mathbf{W}) \qquad \text{(by independence).}
\end{aligned}$$

Recall that the independence assumptions are $U \perp (L, C)|\mathrm{X}, \mathbf{W}, T > L$ and $\mathrm{X} \perp (L, C)|\mathbf{W}$, which imply that $f(T, \delta|\mathrm{X}, \mathbf{W}, L) = f(T, \delta|\mathrm{X}, \mathbf{W})$ for $T > L$ and $p(\mathrm{X}|\mathbf{W}, L) = p(\mathrm{X}|\mathbf{W})$, respectively. This completes the derivation of Equation (2) in the main text.

2 MCAR Simulation Results with Large n

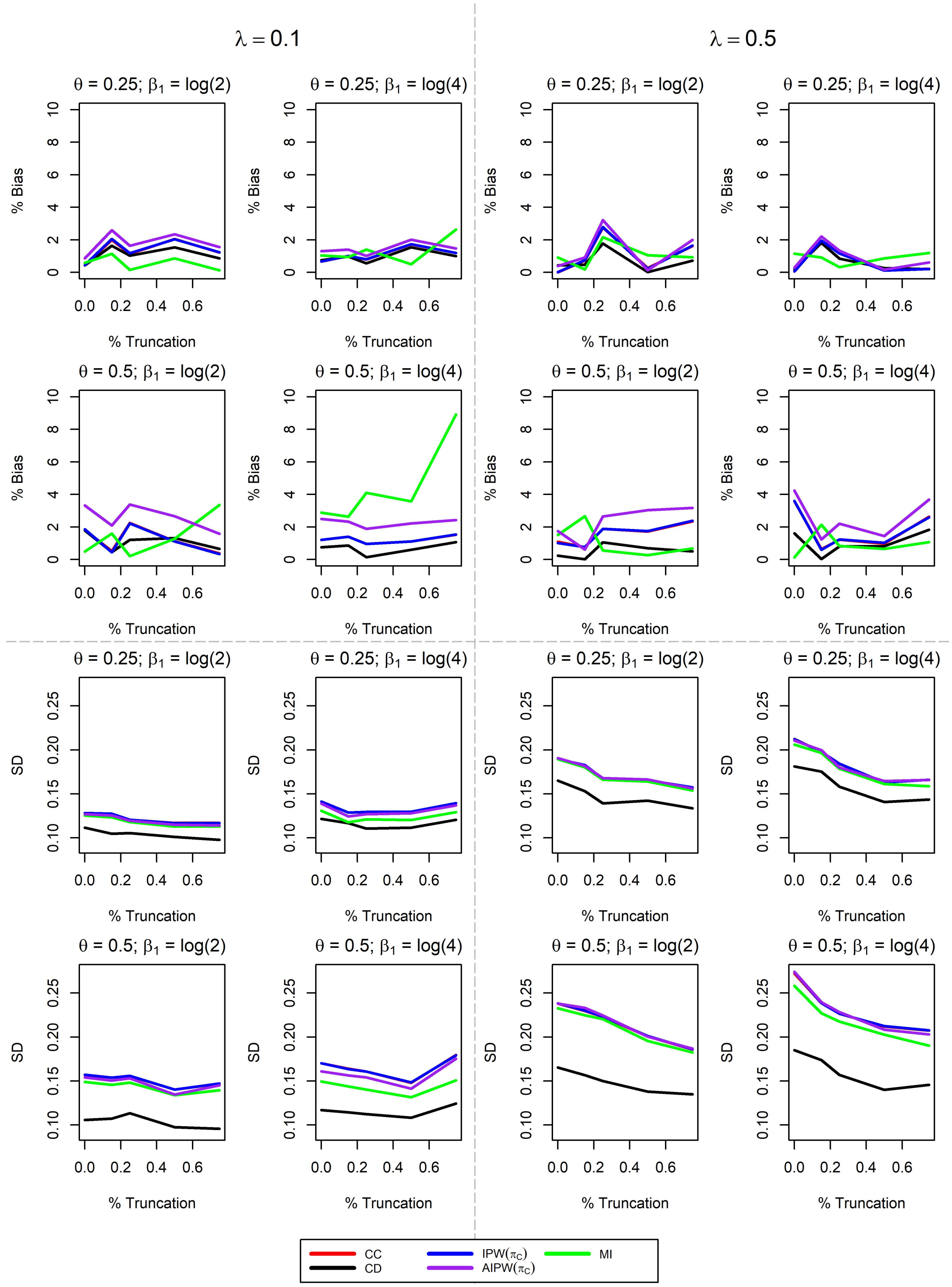

Figure S.1: Extended Simulation Results for MCAR Missingness with $n = 500$. β_1 is the log-hazard ratio of the missing covariate X, λ is the censoring rate and θ is the proportion of data with X missing.

3 δ-Dependent MAR Simulation Results with Large n

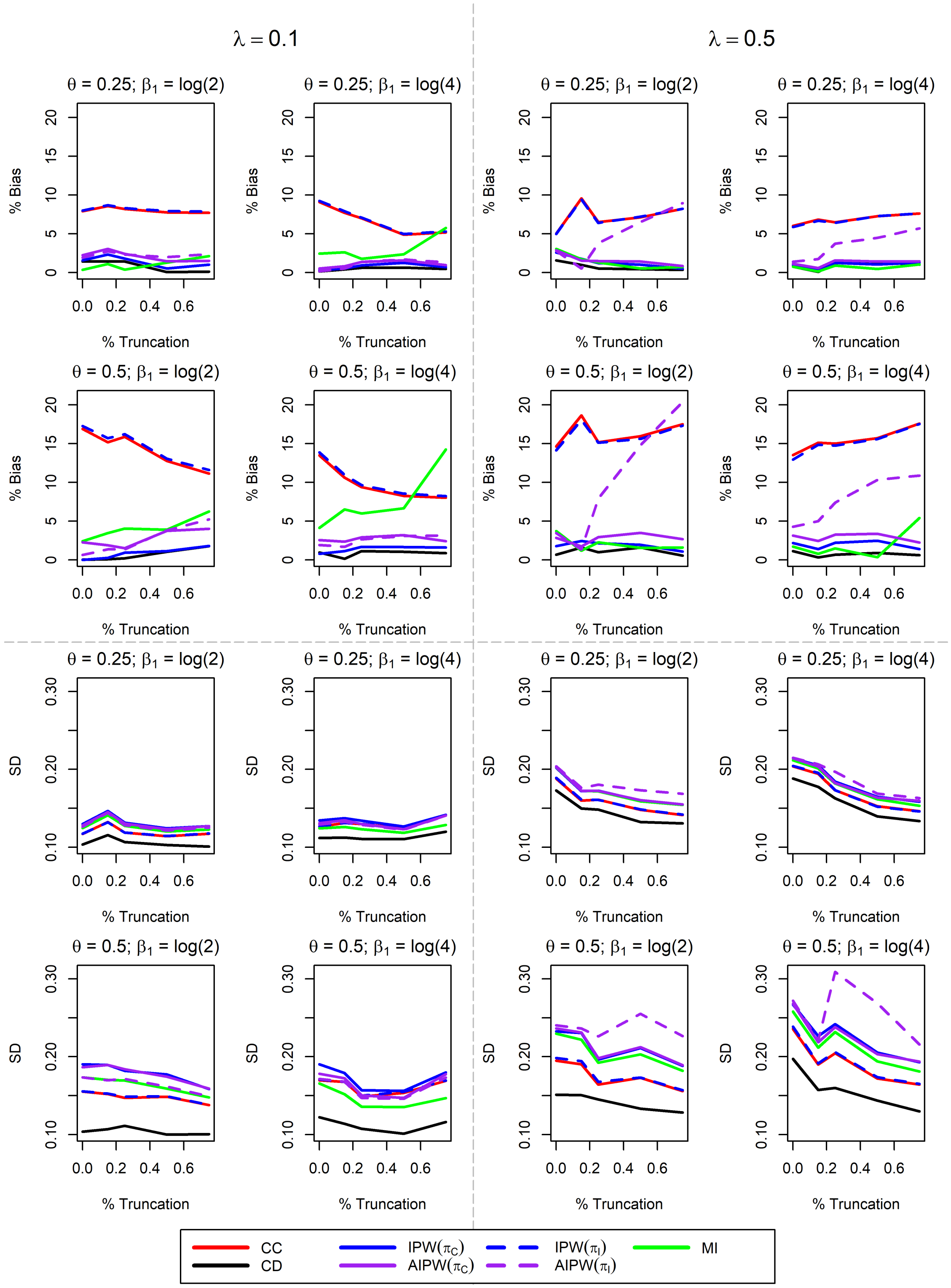

Figure S.2: Extended Simulation Results for δ-Dependent MAR Missingness with $n = 500$. β_1 is the log-hazard ratio of the missing covariate X, λ is the censoring rate and θ is the proportion of data with X missing.

4 T-Dependent MAR Simulation Results with Large n

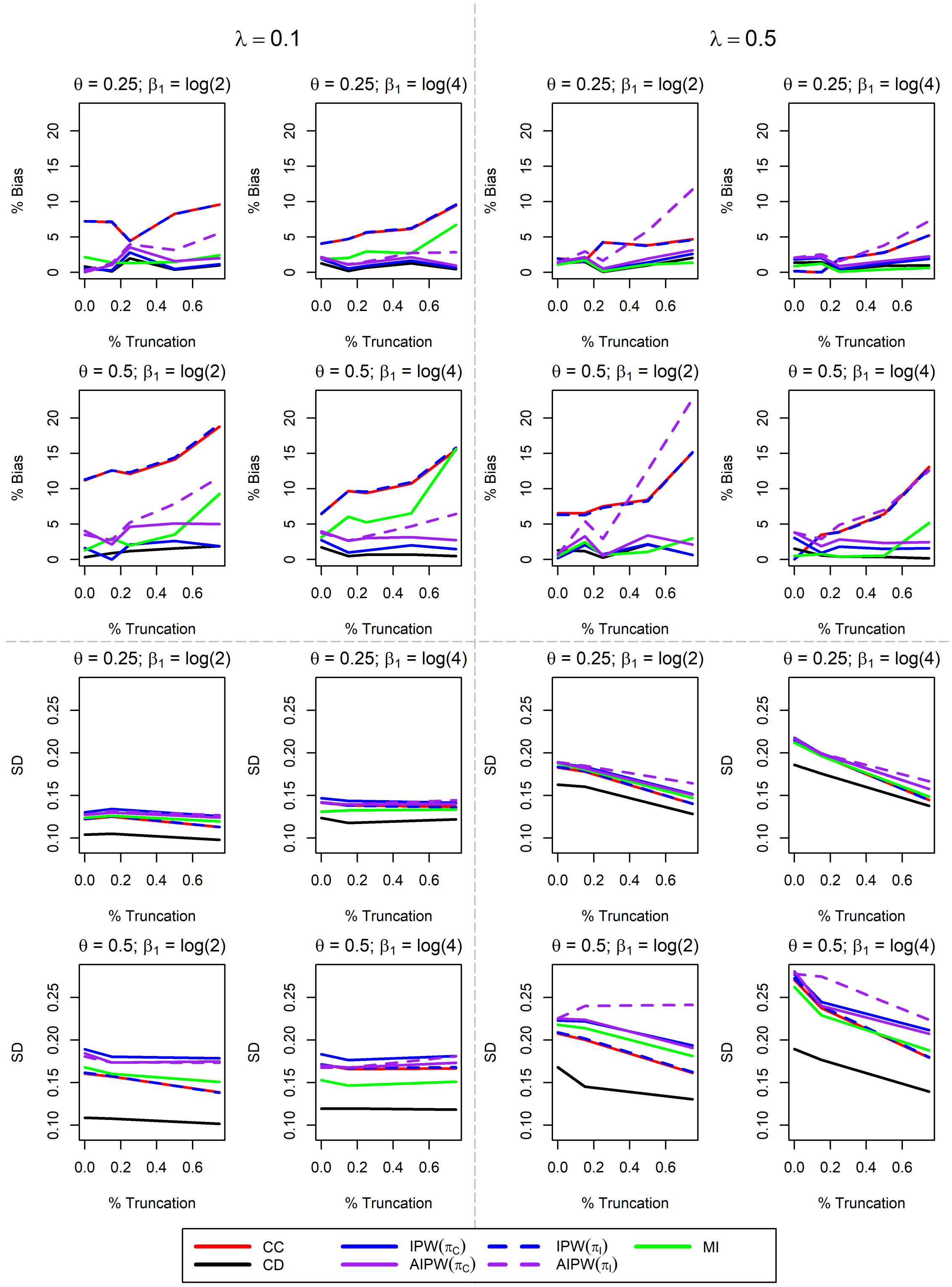

Figure S.3: Extended Simulation Results for T-Dependent MAR Missingness with $n = 500$. β_1 is the log-hazard ratio of the missing covariate X, λ is the censoring rate and θ is the proportion of data with X missing.

5 R Code

```
# R code for CC, IPW, MI, and AIPW methods.

library(survival)
library(mice)
library(nleqslv)

# Get AIPW coefficient estimates for Cox model with single binary missing covariate X
# - For convergence issues, consider increasing the max iterations or using a different
#   initial value
# - Other missing covariate distributions will require a different score in the
#   est_equation() function for the new imputation model, as well as changes to cox score
fit_aipw <- function(W_cox, W_imp, X, event_time, L, delta, ipw,
                     max_iters = 100, min_density = 1e-5){

  # compute estimating function value
  est_equation <- function(coef_ests, hazard){
    coef_imp <- coef_ests[1:p_imp]
    coef_cox <- coef_ests[-(1:p_imp)]

    # calculate imputed X | T,delta,W,L
    fitted_X <- plogis(c(W_imp %*% coef_imp))
    exps_X <- exp(coef_cox[1] * XW[, 1])
    exps_W <- exp(c(XW[, -1] %*% coef_cox[-1]))
    haz <- hazard[match(event_time, hazard[, 1]), 2]
    chaz <- sapply(event_time, FUN = function(etime) sum(hazard[hazard[, 1] <= etime, 2]))
    cox_density0 <- pmax(ifelse(delta == 1, exps_W * haz, 1) * exp(-chaz * exps_W), min_density)
    cox_density1 <- pmax(ifelse(delta == 1, exps_W * exp(coef_cox[1]) * haz, 1) *
                          exp(-chaz * exps_W * exp(coef_cox[1])), min_density)
    norm_const <- cox_density1 * fitted_X + cox_density0 * (1 - fitted_X)
    imputed_X <- ifelse(cox_density1 < min_density, 0, cox_density1 * fitted_X / norm_const)
    imputed_exps <- exps_W * (exp(coef_cox[1]) * imputed_X + (1 - imputed_X))
    imputed_XWexps <- diag(exps_W * exp(coef_cox[1]) * imputed_X) %*% X1W
    imputed_XW <- cbind(imputed_X, XW[, -1])

    # cox model score
    wts1 <- R * ipw * exps_X * exps_W
    wts2 <- (1 - R * ipw) * imputed_exps
    wts3 <- (1 - R * ipw) * exps_W * exp(coef_cox[1]) * imputed_X
    score_cox <- c(t(R * delta * ipw) %*% XW) + c(t((1 - R * ipw) * delta) %*% imputed_XW)
    deriv <- matrix(0, nrow = ncol(XW), ncol = ncol(XW))
    # risk set averages in score and derivative
    for (j in seq_along(unique_etimes)){
      riskindx <- (event_time >= unique_etimes[j]) & (unique_etimes[j] > L)
      match_indx <- event_time == unique_etimes[j]
      n_delta <- sum(delta[match_indx])
      S0 <- sum(riskindx * (wts1 + wts2))
      S1 <- c((t(riskindx * wts1) %*% XW) + (t(riskindx * wts3) %*% X1W))
      score_cox <- score_cox - n_delta * S1 / S0
    }
    score_cox <- score_cox

    # imputation model score
```

```
  score_imp <- c(t((R * ipw * X) + (1 - R * ipw) * imputed_X - fitted_X) %*% W_imp)

  return(c(score_imp, score_cox))
}

n <- nrow(W_cox)
R <- !is.na(X)
X[!R] <- 0
XW <- cbind(X, W_cox)
W_imp <- cbind(1, W_imp)
unique_etimes <- unique(event_time)
X1W <- cbind(1, XW[, -1])
p_imp <- ncol(W_imp)
p_cox <- ncol(XW)

# initialize estimates
coef_imp <- rep(0, p_imp)
coef_cox <- rep(0, p_cox)
hazard <-  cbind(event_time[delta == 1],
                 sapply(event_time[delta == 1], FUN = function(etime){
                   riskindx <- (event_time >= etime) & (etime > L)
                   1 /  sum(riskindx)
                 }))
# alternate between updating coefficients and baseline hazard
for (i in seq_len(max_iters)){
  updated_ests <- nleqslv(x = c(coef_imp, coef_cox),
          fn = function(ests) est_equation(ests, hazard),
          method="Broyden",
          control = list(maxit = 10))
  if ((updated_ests$iter <= 1) & (updated_ests$termcd == 1)){
    break
  }

  coef_imp <- updated_ests$x[1:p_imp]
  coef_cox <- updated_ests$x[-(1:p_imp)]

  # calculate imputed X | T,delta,W,L
  fitted_X <- plogis(c(W_imp %*% coef_imp))
  exps_X <- exp(coef_cox[1] * XW[, 1])
  exps_W <- exp(c(XW[, -1] %*% coef_cox[-1]))
  haz <- hazard[match(event_time, hazard[, 1]), 2]
  chaz <- sapply(event_time, FUN = function(etime) sum(hazard[hazard[, 1] <= etime, 2]))
  cox_density0 <- pmax(ifelse(delta == 1, exps_W * haz, 1) * exp(-chaz * exps_W),
                       min_density)
  cox_density1 <- pmax(ifelse(delta == 1, exps_W * exp(coef_cox[1]) * haz, 1) *
                         exp(-chaz * exps_W * exp(coef_cox[1])), min_density)
  norm_const <- cox_density1 * fitted_X + cox_density0 * (1 - fitted_X)
  imputed_X <- ifelse(cox_density1 < min_density, 0, cox_density1 * fitted_X / norm_const)
  imputed_exps <- exps_W * (exp(coef_cox[1]) * imputed_X + (1 - imputed_X))

  hazard <- cbind(event_time[delta == 1],
                  sapply(event_time[delta == 1], FUN = function(etime){
                    riskindx <- (event_time >= etime) & (etime > L)
                    1 /  sum(riskindx * (R * ipw * exps_X * exps_W +
```

```
                                            (1 - R * ipw) * imputed_exps))
                    }))
    }

  return(list(coef = coef_cox, converged = (i < max_iters) & (updated_ests$termcd == 1)))
}

# Get multiple imputation estimates for Cox model with single binary missing covariate X
# - Other missing covariate distributions will require a change to the mice() function call
#   to use a different model
fit_mi <- function(W_cox, W_imp, X, event_time, L, delta, n_imputations = 100){
  # compute hazard
  hazard <-  basehaz(coxph(Surv(L, event_time, delta) ~ 1))
  chaz <- hazard[match(event_time, hazard[, 2]), 1]
  chaz[event_time < min(hazard[, 2])] <- 0
  chaz[event_time > max(hazard[, 2])] <- max(hazard[, 1])

  # imputation model predictors
  R <- ifelse(!is.na(X), 1, 0)
  predictors <- as.data.frame(cbind(delta, chaz, W_imp, diag(chaz) %*% W_imp, X))
  colnames(predictors) <- c("delta", "haz", colnames(W_imp),
                            paste0("haz", colnames(W_imp)), "X")

  # generate estimates from imputed data
  y <- Surv(L, event_time, delta)
  imps <- suppressWarnings(mice(predictors, m = n_imputations, method = "logreg",
                                print = FALSE, maxit = 1))
  fits <- with(imps, coxph(y ~ W_cox + X))
  results <- summary(pool(fits, dfcom = nrow(predictors)))

  return(results)
}

# Data
L # left truncation times
event_time # observed event time
delta # event indicator
R # non-missingness indicator (0/1)
W_cox # design matrix for non-missing covariates in cox model
W_pi # design matrix for missingness model (no intercept)
W_imp # design matrix for non-missing covariates in imputation model (no intercept)
X # covariate vector subject to missingness

# Fit missingness model for inverse probability weights
missingness_fit <- glm(R ~ W_pi, family = binomial())
ipw <- 1 / predict(missingness_fit, type = "response")

# cc
cc_fit <- coxph(Surv(L, event_time, delta) ~ cbind(X, W_cox))

# ipw
ipw_fit <- coxph(Surv(L, event_time, delta) ~ cbind(X, W_cox), weights = ipw)

# aipw (with bootstrap std error)
```

```
aipw_fit <- fit_aipw(W_cox, W_imp, X, event_time, L, delta, ipw)
boot_fn <- function(n_boot){
  res <- matrix(NA, nrow = n_boot, ncol = 2)
  for (j in seq_len(n_boot)){
    indx <- sample.int(length(X), replace = TRUE)
    resp <- R[indx]
    preds <- W_pi[indx, ]
    missingness_fit <- glm(resp ~ preds, family = binomial())
    ipw <- 1 / predict(missingness_fit, type = "response")

    res[j, ] <- tryCatch(
      {fit <- fit_aipw(W_cox[indx, ], W_imp[indx, ], X[indx], event_time[indx],
                       L[indx], delta[indx], ipw)
      c(fit$coef[1], ifelse(fit$converged, 1, 0))},
      error = function(err) c(NA, 0))
  }
  res
}
boot_ests <- boot_fn(550)

# mi
mi_fit <- fit_mi(W_cox, W_imp, X, event_time, L, delta, n_imputations = 100)

# table of results
ests <- data.frame(method = c("CC", "IPW", "AIPW", "MI"),
                   coef = c(coef(cc_fit)[1], coef(ipw_fit)[1], aipw_fit$coef[1],
                            mi_fit$estimate[3]),
                   se = c(sqrt(diag(vcov(cc_fit))[1]), sqrt(diag(vcov(ipw_fit))[1]),
                          mad(boot_ests[boot_ests[, 2] == 1, 1]), mi_fit$std.error[3]))
ests$pvalue <- 2 * pnorm(abs(ests$coef / ests$se), lower.tail = FALSE)
knitr::kable(ests, digits = 3)
```
